# Maternal PTSD symptoms and sensitivity during caregiving in early postpartum: The moderating role of resting and reactive RSA in a trauma-exposed sample

**DOI:** 10.1017/S0033291725102432

**Published:** 2025-11-11

**Authors:** Abigail Powers, Rebecca Lipschutz, Elizabeth McAfee, Catherine Abrams, David O’Banion, Vasiliki Michopoulos, Patricia Brennan, Jennifer Stevens

**Affiliations:** 1 Department of Psychiatry and Behavioral Sciences, Emory University School of Medicine, Atlanta, GA, USA; 2 Department of Pediatrics, Emory University School of Medicine, Atlanta, GA, USA; 3 Department of Gynecology and Obstetrics, Emory University School of Medicine, Atlanta, GA, USA; 4 Emory National Primate Research Center, Atlanta, GA, USA; 5 Department of Psychology, Emory University, Atlanta, GA, USA; 6 Atlanta VA Medical Center, Atlanta, GA, USA

**Keywords:** autonomic function, maternal sensitivity, postpartum, PTSD, respiratory sinus arrhythmia

## Abstract

**Background:**

Impaired maternal sensitivity may be a risk pathway linking maternal posttraumatic stress symptoms (PTSS) to adverse child outcomes. Respiratory sinus arrhythmia (RSA), a psychophysiological marker of emotion dysregulation, may be a key factor in how PTSS influence maternal sensitivity. Yet, these associations remain untested in early infancy. The current study tested maternal resting RSA and RSA reactivity to caregiving as moderators of the association between maternal PTSS and maternal sensitivity in trauma-exposed mothers.

**Methods:**

Seventy-seven mother–infant dyads (maternal *M_age_* = 30.06 years, infant *M_age_* = 9.53 weeks) were recruited from the community and an urban public hospital setting. Mothers reported on PTSS and engaged in a caregiving task; maternal sensitivity was coded. RSA was measured at rest and in response to the task. Generalized linear models for ordinal outcomes analyses examined the moderating effect of resting RSA and RSA reactivity (decrease in RSA) on the association between PTSS and maternal sensitivity.

**Results:**

The association between maternal PTSS and sensitivity was significantly moderated by resting RSA (*B(SE) =* 0.03(0.01), *p* = .033, and RSA reactivity, *B(SE) =* 0.03(0.01), *p* = .022.

Maternal PTSS was negatively associated with maternal sensitivity only among mothers with higher resting RSA (+1SD above mean), *B(SE)* = −0.05(0.02), *p* = .030, and with greater RSA reactivity (−1SD below mean RSA reactivity scores), *B(SE)* = −0.06 (0.02), *p* = 0.021.

**Conclusions:**

A tendency toward autonomic overregulation and heightened physiological reactivity may serve as relevant factors influencing how PTSS leads to maladaptive parenting behavior in early postpartum.

## Introduction

Posttraumatic stress disorder (PTSD) is a debilitating, stress-related psychiatric illness that can occur following trauma exposure and affects 8.3% of the nation’s population (Kilpatrick et al., 2013). Some individuals are more vulnerable to PTSD than others, with women at two-to-three times the risk of developing PTSD compared to men (Tortella-Feliu et al., 2019). Importantly, the detrimental effects of posttraumatic stress symptoms (PTSS) can be perpetuated across generations, and early parenting behaviors are an important risk pathway linking maternal PTSS to adverse child outcomes (Amstadter et al., 2024). Because of the potential detrimental impact of PTSS in the postpartum period, understanding how PTSS are associated with parenting difficulties in early postpartum and examining individual-level factors that may influence these associations is of significant value.

Early interactions with mothers are the building blocks for socioemotional development in children (Frenkel & Fox, 2014). For the scope of this study, we focus on mothers but acknowledge the important role that fathers and other caregivers play in the development and wellbeing of infants. Maternal sensitivity, that is warmth and attentive, timely, and consistent parental responsiveness toward infant signals, is a critical component of the maternal caregiving system (Bell & Ainsworth, 1972; Lohaus, Keller, Ball, Elben, & Voelker, 2001) and is linked to a wide range of positive child outcomes (Montealegre-Ramón, Martínez-Fuentes, Pérez-López, & Sierra-García, 2024). Maternal sensitivity can even buffer against the negative effects of mental health symptoms (Kaplan, Burgess, Sliter, & Moreno, 2009). Parental responsiveness during lab-based stressors has been linked to reduced behavioral and physiological reactivity in infants (Haley & Stansbury, 2003), suggesting the value of maternal sensitivity in the context of distress. Examining maternal sensitivity in the context of everyday caregiving tasks common in infancy (e.g., diaper change) may be of value as this can be a time when distress may arise and the need for sensitivity is key.

Research generally supports the link between maternal PTSS and lower maternal sensitivity in early development, although findings are mixed, and direct examination in the context of caregiving remains unstudied. Results from a latent profile analysis study with postpartum mothers showed that class membership in the ‘stable high PTSS’ through 4 months postpartum was associated with lower maternal sensitivity during free play, independent of other internalizing symptoms (Rousseau, Feldman, Shlomi Polachek, & Frenkel, 2023). Similarly, maternal PTSS was related to lower maternal sensitivity during free play at 12–42 months postpartum in a sample of White intimate partner violence (IPV) exposed mothers (Schechter et al., 2015). However, in a racially diverse sample of mothers at 12 months postpartum, PTSS were not related to maternal sensitivity during free play or a stressor (Huffhines, Coe, Busuito, Seifer, & Parade, 2022). A study of mothers with pre-term infants at 12 months postpartum found that PTSS were related to greater maternal sensitivity during free play (Elansary et al., 2022). Given mixed findings, it is critical to better understand the association between maternal PTSS and maternal sensitivity behavior, particularly in the first few months postpartum when risk pathways may first develop.

It is possible that individual-level factors contribute to the variability in the PTSS-maternal sensitivity connection, and so it is important to identify potential moderators that might put some women with PTSS at risk for less sensitive parenting behaviors. Emotion dysregulation, a transdiagnostic factor in psychopathology (Aldao, Gee, De Los Reyes, & Seager, 2016; Beauchaine & Cicchetti, 2019), plays a major role in PTSS (Powers, Cross, Fani, & Bradley, 2015; Seligowski, Lee, Bardeen, & Orcutt, 2015). Parenting, in the context of infant demands or distress, places high needs on emotion regulation capacity, and the presence of emotion dysregulation can have a substantial impact on parental capacity (Powers et al., 2022). Respiratory sinus arrhythmia (RSA) is a psychophysiological marker of emotion dysregulation (Beauchaine, 2015) and may be a key factor in how PTSS influence early maternal caregiving behavior based on variability in maternal physiological regulation capacity and response to caregiving, but this remains untested.

RSA reflects the naturally occurring variation in heart rate synchronized with respiration and is an indirect measure of parasympathetic nervous system (PNS) activity (Berntson et al., 1997). Typically, the PNS is engaged at rest to regulate cardiac functioning and disengages or ‘withdraws’ in response to environmental or emotional stressors. RSA can be measured at rest (reflecting physiological regulation capacity) and in response to emotion evocation or stress (reflecting physiological reactivity). RSA reactivity is the change in RSA from rest in response to a situation, typically decreasing in response to stress and representing adaptive parasympathetic withdrawal that facilitates active coping (Porges, 2007). Greater decreases in RSA in response to stress indicate greater RSA reactivity (Porges, 2007), whereas lower or blunted RSA reactivity reflects minimal or absent RSA change. Higher resting RSA represents greater capacity for physiological and emotion regulation (Pinna & Edwards, 2020). Both low resting RSA and excessive RSA reactivity (that is exaggerated decreases in RSA in response to stress) signify impaired top-down emotion-regulatory processes (Beauchaine, 2015; Porges, 2007) and have consistently been found across a wide range of psychopathology (Beauchaine, 2015), including PTSD (Campbell & Wisco, 2021; Campbell, Wisco, Silvia, & Gay, 2019; Schneider & Schwerdtfeger, 2020). Alternatively, blunted RSA reactivity (i.e., minimal or no change in RSA in response to stress) may be present in those with psychopathology in the context of social stress (Shahrestani, Stewart, Quintana, Hickie, & Guastella, 2014).

Flexibility of the autonomic nervous system in response to parental demands is key to maternal sensitivity. Lower resting RSA (i.e., reduced physiological regulation capacity) is related to less sensitive parenting (Musser, Ablow, & Measelle, 2012). In contrast, moderate RSA reactivity (i.e., a decrease in RSA) can support adaptive responding to infant distress and greater maternal sensitivity toward the infant (Joosen et al., 2013; Leerkes, Su, Calkins, Supple, & O’Brien, 2016). However, the adaptiveness of RSA reactivity is context-dependent (Augustine & Leerkes, 2019). In caregiving situations involving high infant distress, greater RSA reactivity may be adaptive since it supports a physiological response in mothers that enables quick responding, while in lower arousal situations, less RSA reactivity may be adaptive because it facilitates more social engagement (Augustine & Leerkes, 2019; Ravindran, McElwain, Berry, & Kramer, 2022). Thus, flexible modulation of mothers’ physiological reactivity across contexts is key for adaptive caregiving interactions. How maternal physiology relates to maternal sensitivity in the context of caregiving in infancy remains unknown. Infancy is an especially important time to understand maternal psychophysiological function (or dysfunction) because infants rely on their caregivers to help them regulate their distress (Luecken & Lemery, 2004; Taipale, 2016). Impairment in maternal physiological functioning may be a key effect modifier in the link between maternal PTSS and maternal sensitivity, making it a potential target for intervention programs that would reduce adverse infant and child outcomes (Cook, Ayers, & Horsch, 2018; Enlow et al., 2011).

Unfortunately, the research on maternal PTSS, RSA reactivity, and maternal sensitivity is almost nonexistent. To our knowledge, only one study has examined relations between maternal PTSS, maternal RSA reactivity, and parenting behaviors, and it was not with infants. Gurtovenko and Katz (2020) explored how RSA reactivity moderated the relation between PTSS and parenting behaviors in a sample of IPV survivors and their children ages 6 to 12. Results showed that maternal PTSS was related to more non-supportive parenting reactions when discussing a recent disagreement, when mothers exhibited less RSA reactivity, whereas greater RSA reactivity (i.e., greater decrease in RSA) buffered the association between PTSS and non-supportive parenting. Thus, RSA reactivity in the context of parenting may have a risk or protective effect against the adverse effects of PTSS on parenting behavior, but how this works in early development or in a caregiving context among trauma-exposed mothers and their infants is undetermined.

To address major gaps in research, the current study will test the moderating effects of maternal resting RSA and RSA reactivity to a caregiving task on the relation between maternal PTSS and maternal sensitivity in a sample of trauma-exposed mothers and their 6- to 10-week-old infants. Given the research on the association between PTSS and RSA to date, we hypothesize that maternal PTSS will be related to lower maternal sensitivity in the context of (1) lower resting RSA and (2) greater RSA reactivity (evidenced as a greater decrease in RSA). Examining how PTSS influence maternal sensitivity within the context of physiological regulation during a caregiving task with young infants will help to clarify individual-level processes contributing to the intergenerational transmission of trauma-related stress.

## Methods

### Procedure

Participants were recruited for an NIH-funded longitudinal study of trauma-exposed mothers and their infants. Participants were recruited and enrolled via social media advertisements and from the obstetrics clinic at an urban, publicly funded hospital. Mothers were assessed for study eligibility by phone. Inclusion criteria: aged 18+, trauma-exposed (based on DSM-5 criterion A), able to provide informed consent, and able to undergo a magnetic resonance imaging (MRI) scan; infants had to be less than 8 weeks old, born at full term (>37 weeks), and free of congenital or heart conditions. Exclusion criteria included the presence of active psychotic symptoms. Exposure to criterion A trauma (i.e., exposure to actual or threatened death, serious injury, or sexual violence) was screened on the eligibility phone call and confirmed during a clinical interview with a study clinician. All participants provided informed consent approved by the university’s Institutional Review Board and the hospital’s Research Oversight Committee. Procedures were conducted in accordance with IRB guidelines and regulations in accordance with the Declaration of Helsinki. Participants were compensated for their time, and transportation to/from visits was provided as needed. The current study included the first in-person visit (~6–10 weeks postpartum) where mothers completed assessments on psychological symptoms and engaged in a dyadic behavioral interaction task while physiological data were recorded. Participants with coded behavioral data were included in the study sample (*N* = 77).

### Participants

This study included a diverse sample of postpartum mothers (*N* = 77, *M*_*mother* age_ = 30.06 years, *SD_mother age_* = 5.72 years; 61.0% Black) and their infants (*M*_*baby* age_ = 9.53 weeks, *SD_baby age_* = 1.54 weeks). Demographic details are provided in Table 1.

**Table 1.** Sample characteristics (*n* = 77)

	% (*n*)
Race	
*African American or Black*	61% (47)
*Asian*	5% (4)
*White*	26% (20)
*More than one race*	5% (4)
*Other*	3% (2)
Currently employed	58% (45)
Insurance status	57% (44)
*No insurance*	3% (2)
*Medicaid*	55% (42)
*Private insurance*	42% (31)
Parity	
*Primiparous*	44% (34)
*Multiparous*	56% (43)
Education level	
*Less than 12th grade*	6% (5)
*High school graduate/GED*	20% (15)
*Some college/technical school*	22% (17)
*College/technical school graduate*	31% (24)
*Graduate degree*	21% (16)
Current relationship status	
*Single, never married*	47% (36)
*Domestic partner*	4% (3)
*Married*	45% (35)
*Divorced*	3% (2)
*Widowed*	1% (1)

### Measures

The PTSD Checklist for DSM-5 (PCL-5) (Blevins, Weathers, Davis, Witte, & Domino, 2015) is a 20-item self-report questionnaire used to assess PTSD symptoms within the past month. Participants rate each item on a scale from 0 (*not at all*) to 4 (*extremely*); total scores reflect overall PTSD symptom severity. The PCL-5 has demonstrated strong psychometric properties across multiple samples (Blevins et al., 2015; Bovin et al., 2016; Mekawi et al., 2022). Cronbach’s alpha in this sample was .94.

The Life Events Checklist (LEC) (Weathers et al., 2013) is a 14-item screen that assesses exposure to potentially traumatic events. An additional item was added to assess for life-threatening pregnancy/birth-related experiences. Participants indicate the level of exposure to the traumatic or stressful experience (happened to me, witnessed it, learned about it). Questions from the LEC extended version were included to assess (1) if the event was life-threatening to the person or others or (2) involved serious injury to the person or others. The number of types of events witnessed or experienced was summed to create a total score for trauma load.

Demographics and potential covariates were measured. Mothers reported on education, monthly income, health insurance status, employment, marital status, parity, current smoking status, current psychiatric medication use, and infant sex. Maternal depression, measured with the Edinburgh Postnatal Depression Scale (Cox, Holden, & Sagovsky, 1987) was assessed as a potential covariate. Infant negativity to parent, coded during the caregiving task, was also assessed as a potential covariate (see Supplementary Materials).

### Behavioral caregiving task

Mothers engaged in a caregiving task with their infant; this task may serve as a stressor for some infants (Mörelius, Nelson, & Gustafsson, 2007). Mothers were instructed to remove infant clothing, change their diaper, apply lotion, and redress their infant. The order of these activities remained constant across infants. The task was video-recorded.

### RSA data collection and processing

Dyads first participated in a 2-minute resting baseline followed by continuous recording during the caregiving task. Electrocardiogram (ECG) and impedance cardiography (respiration) signals were recorded using MindWare mobile units, and respiration was monitored to ensure values were within the valid range for RSA calculation. RSA was derived as high-frequency heart rate variability (.15–.40 Hz for mothers) using spectral analysis procedures (Fracasso, Porges, Lamb, & Rosenberg, 1994). The natural log of derived values was used. Data were analyzed in Mindware HRV 3.0 software using 60-second epochs, with no more than 15 seconds of artifact for any one epoch; epochs were visually inspected and corrected for artifacts or mismarked peaks by trained research staff. Additional details are in the Supplementary Materials.

Baseline RSA was averaged across both epochs (120 seconds total), and caregiving task RSA was averaged across epochs for up to the first 8 minutes of data. The caregiving task was not time-limited, so task duration varied across participants (*M* = 4.85 minutes, range = 1.9–15 minutes). Three participants had data over eight minutes and these epochs were not included. Four participants were missing RSA data (3 technical issues; 1 heart arrhythmia). RSA reactivity scores were calculated as the difference between average task RSA and baseline RSA; negative values reflect a decrease in RSA in response to the caregiving task from baseline or greater RSA reactivity, and positive values an increase in RSA in response to the caregiving task from baseline (less RSA reactivity).

### Maternal sensitivity coding

Maternal sensitivity was coded based on video recordings of the caregiving task. Coding was adapted from the NICHD Study of Early Child Care, which has demonstrated similar psychometric properties across ethnoracially diverse dyads (Engel et al., 2021; Fuligni & Brooks-Gunn, 2013). Maternal sensitivity was defined as a mother’s ability to cultivate a child-centered interaction, remain highly attentive and respond appropriately to her infant’s needs or distress during the interaction. Sensitivity was rated on a 1 (*low sensitivity*) to 4 (*high sensitivity*) to infant cues. Raters were blind to family characteristics and did not engage in study visits. Raters were trained and met reliability prior to rating interactions for this study. To ensure ongoing reliability, ~20% of the videos were coded by a second rater. Sensitivity ratings demonstrated strong interrater reliability (*ICC* = .89).

### Data analysis

Distributions and outliers of key variables were examined. Level of skewness and kurtosis in this sample fell within acceptable parameters (Tabachnick & Fidell, 2001). Descriptive statistics and bivariate Pearson correlations between PTSS, maternal sensitivity, resting RSA and RSA reactivity were examined (Table 2). Maternal age, trauma load, health insurance status (None or Medicaid/Private insurance), parity (primiparous/multiparous), current smoking status (Yes/No), current psychiatric medication use (Yes/No), postpartum depression symptoms, infant negativity toward parent (None/Some negativity), and infant sex (Male/Female) were examined as covariates of interest. Only parity was significantly correlated with maternal sensitivity at *p* < .05 and was subsequently included in all models as a covariate. All statistical analyses were conducted using Jamovi 2.6.

**Table 2.** Correlations and descriptives of key study variables

	PTSS	Maternal sensitivity	Resting RSA	RSA reactivity
Maternal sensitivity	−0.16	–		
Resting RSA	0.001	−0.02	–	
RSA reactivity	−0.01	0.15	−0.64***	–
M(SD)	17.77 (15.64)	2.97 (1.01)	5.27 (1.07)	−0.65 (1.13)
Range	0.00–55.00	1.00–4.00	2.71–7.37	−3.39–1.88

*Note.* ***p < .001. PTSS = Posttraumatic stress symptoms measured with PCL5. RSA reactivity = RSA_caregiving_ – RSA_baseline_.

Data were analyzed using generalized linear models (GLMs) for ordinal outcomes, given that maternal sensitivity scores were measured using an ordinal rating scale (coded 1–4). Although smaller effects may be underpowered, the current sample size (*N* = 77) aligns with established recommendations for detecting moderate interaction effects in regression frameworks (Fritz & MacKinnon, 2007). Simulation-based tutorials for ordinal logistic regression (Gambarota & Altoè, 2024) indicate that analogous effect sizes should also be detectable with ordinal outcomes. Primary analyses examined the associations between maternal PTSS, RSA, and maternal sensitivity. The primary predictors in the model were maternal PTSS, maternal RSA (resting and reactivity), and the interaction between PTSS and maternal RSA measures. Separate models were examined for resting RSA and RSA reactivity. Significant interactions were probed using simple slopes analyses to examine resting RSA and reactivity as moderators of the association between PTSS and maternal sensitivity. Simple slopes were plotted and estimated at three levels of RSA measures (−1SD, mean, 1SD). Model assumptions, including normality of residuals and multicollinearity, were assessed. In these models for ordinal outcomes, intercepts are the log-odds of selecting a maternal sensitivity rating at that level or below. The regression coefficient (B) describes the increase in log-odds of selecting a maternal sensitivity rating at that level or below associated with a one unit increase in the predictor. In other words, the regression coefficient indicates how much the predictor increases the probability of choosing a higher level for maternal sensitivity, so taking a step up on the ordinal scale.

## Results

**Figure 1.**
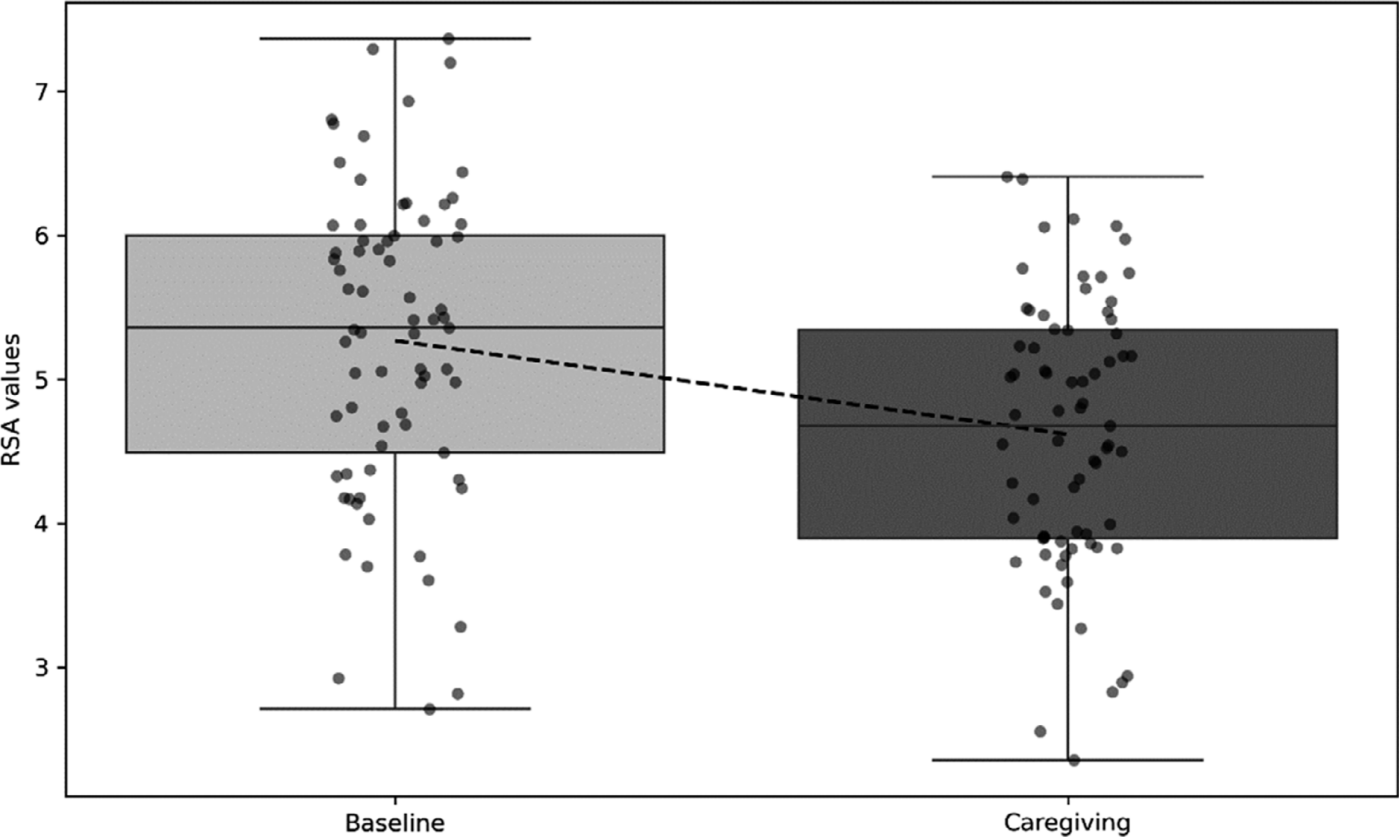
Mean RSA values across baseline and caregiving task.

Descriptive and bivariate correlations for key study variables are shown in Table 2. Baseline RSA was significantly negatively correlated with RSA reactivity (*r =* −0.64, *p* < .001). RSA decreased from the baseline, M(SD) = 5.27 (1.07), to the caregiving task, M(SD) = 4.62 (0.94), *t*(72) = 4.91, *p* < .001 (see Figure 1).

### Resting RSA

**Figure 2.**
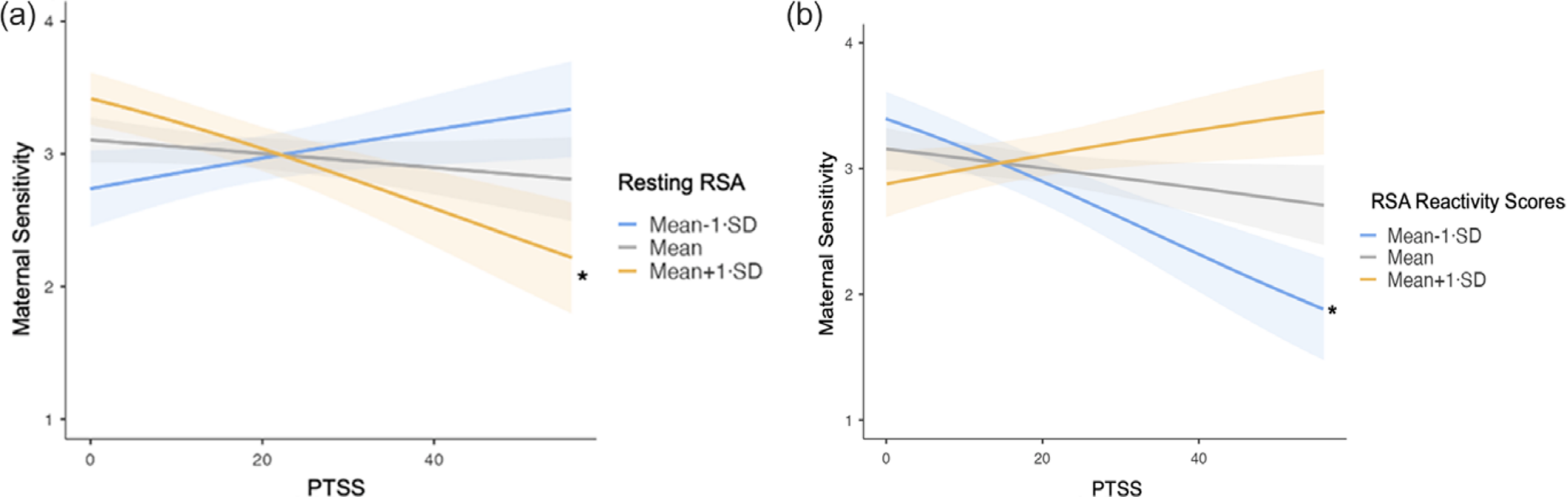
Resting RSA and RSA reactivity moderate associations between maternal posttraumatic stress symptoms (PTSS) and maternal sensitivity. Moderators are plotted at ±1SD of mean RSA values. (2a) There was a significant negative association between PTSS and maternal sensitivity for mothers with higher resting RSA values (+1SD) as indicated with an asterisk. (2b) RSA reactivity = RSA_caregiving_ – RSA_baseline._ Negative RSA reactivity scores (−1SD) reflect a decrease in RSA (greater RSA reactivity) and positive RSA reactivity score (+1SD) indicate an increase in RSA (less RSA reactivity) in response to the caregiving task. There was a significant negative association between PTSS and maternal sensitivity for mothers with greater RSA reactivity (negative RSA reactivity scores (−1SD)) as indicated with an asterisk.

Model results are shown in Table 3. The interaction between PTSS and resting RSA was significant, *B(SE) =* 0.03(0.01), *p* = .033. Simple effects analysis examined and plotted the interaction at one SD above and below the mean for resting RSA values. As shown in Figure 2, at one SD above the mean of resting RSA, maternal PTSS was negatively associated with maternal sensitivity, *B(SE)* = −0.04(0.02), *p* = .032. There were no significant associations between PTSS and maternal sensitivity at average or above average levels of resting RSA scores (*p*s > .31). This finding contradicts our prediction in Hypothesis 1. Specifically, higher PTSS was linked to lower maternal sensitivity in mothers with higher resting RSA, whereas we expected this association to emerge in mothers with lower resting RSA. All other main effects and predictors were non-significant (ps > .05).

**Table 3.** Generalized linear models for ordinal outcomes results

	*B*	*SE*	*Exp(B)*	*p*
**Resting RSA**				
Intercept 1|2	−2.34	0.40	0.10	<.001
Intercept 2|3	−0.92	0.27	0.40	<.001
Intercept 3|4	0.42	0.25	1.53	.092
PTSS	−0.01	0.01	0.99	.475
Parity	−0.76	0.47	0.47	.103
Resting RSA	0.13	0.22	1.14	.565
PTSS× resting RSA	−0.03	0.01	0.97	.033
**RSA reactivity**				
Intercept 1|2	−2.46	0.44	0.09	<.001
Intercept 2|3	−0.98	0.30	0.37	.001
Intercept 3|4	0.35	0.28	1.42	.207
PTSS	0.01	0.02	1.01	.717
Parity	−0.65	0.48	0.52	.177
RSA reactivity	0.10	0.21	1.11	.628
PTSS×RSA reactivity	0.03	0.01	1.03	.022

*Note.* PTSS = Posttraumatic stress symptoms. Intercepts are the log-odds of selecting a maternal sensitivity rating at that level or below. Parity coded as 0 = no other children, 1 = has prior children. RSA reactivity = RSA_caregiving_ – RSA_baseline_.

### RSA reactivity

Model results are shown in Table 3. The interaction between PTSS and RSA reactivity was significant, *B(SE) =* 0.03(0.01), *p* = .022. Simple effects analysis examined and plotted the interaction at one SD above and below the mean for RSA reactivity scores. As shown in Figure 2, at one SD below the mean of RSA reactivity (negative RSA values, indicating a decrease in RSA and greater RSA reactivity), maternal PTSS was negatively associated with maternal sensitivity, *B(SE)* = −0.05 (0.02), *p* = 0.014. In line with Hypothesis 2, for mothers with greater RSA reactivity (heightened parasympathetic withdrawal during caregiving), higher levels of PTSS were associated with lower maternal sensitivity. There were no significant associations between PTSS and maternal sensitivity at average or above average levels of RSA reactivity scores (*p*s > .28). All other main effects and predictors were non-significant (*ps* > .05).

## Discussion

This is the first study to examine the association between maternal PTSS and maternal sensitivity during caregiving in the early stages of postpartum (<3 months) and to test how maternal parasympathetic regulation capacity and response to a caregiving task moderate this association. RSA, a parasympathetic index of heart rate variability, is typically reflective of greater stress regulatory capacity and flexibility in responding to environmental demands (Berntson et al., 1997; Porges, 2007) and thus is a critical component to how PTSS may affect caregiving behaviors. Our findings highlight parasympathetic regulation as an important effect modifier in the PTSS-impaired maternal sensitivity pathway in trauma-exposed mothers and their infants. Differences in results based on baseline regulatory capacity (i.e., resting RSA levels), and physiological responsiveness to caregiving (i.e., RSA reactivity) have unique clinical implications and both offer valuable insights into how to contextualize maternal PTSS-related risks on parenting in the earliest stages of development.

In examining the specific moderating effect of mothers’ underlying physiological regulation capacity (i.e., resting RSA levels), results showed that the relation between higher PTSS and lower maternal sensitivity was only significant in the context of higher resting RSA. Contrary to our hypothesis, our finding suggests that we only see a major impact of PTSS on maternal sensitivity in the postpartum context for trauma-exposed mothers who appear to have a physiological tendency toward higher autonomic regulation. This is interesting given that we expected the opposite since, in general, low parasympathetic tone is seen as a marker of stress vulnerability and is related to both PTSS (Campbell et al., 2019) and less sensitive parenting (Musser et al., 2012). It is possible that higher parasympathetic tone among those with higher PTSS reflects a tendency toward overregulation or disengagement; the presence of physiological overregulation could make it difficult to actively engage in caregiving behavior for mothers with PTSS and may signal a form of disengagement from oneself or the environment (Carlson, Dalenberg, & McDade-Montez, 2012). Dissociative symptoms are not assessed as part of the PCL-5, the measure of PTSS in the current study, and so we cannot determine if dissociative symptoms were present among the mothers that show heightened resting RSA levels during the caregiving task and may be an area for future research. Importantly, both average and lower resting RSA within this sample appeared to have a buffering effect on the relation between PTSS and maternal sensitivity and it will be valuable to examine what that might mean for infant outcomes moving forward.

Regarding moderating effects of mothers’ physiological responsiveness in the context of caregiving (i.e., RSA reactivity), our finding that higher levels of PTSS are associated with lower maternal sensitivity only in the context of greater RSA reactivity fits with prior research showing PTSS are related to excessive RSA reactivity, broadly (Campbell et al., 2019; Campbell & Wisco, 2021; Fonkoue et al., 2020; Schneider & Schwerdtfeger, 2020). Moderate levels of RSA reactivity (that is parasympathetic withdrawal) to stress can be adaptive, but heightened withdrawal (that is hyperreactivity) is seen as maladaptive (Beauchaine, 2015). While RSA reactivity has been positively associated with maternal sensitivity in healthy mothers (Joosen et al., 2013; Leerkes et al., 2016), our results suggest that over-mobilization of physiological resources during caregiving may be related to less maternal sensitivity for trauma-exposed mothers with high levels of PTSS. Specifically, significant downregulation of the parasympathetic nervous system (and sympathetic or ‘fight or flight’ activation) in response to caregiving may get in the way of a mother’s ability to appropriately respond to infant needs in the moment when PTSS are present, thus negatively affecting maternal sensitivity. Individuals with PTSS often show an interpretation bias toward threat (Arditte Hall & Arditte, 2024) and it is possible that mothers with PTSS and excessive physiological reactivity experience infant distress (or even a neutral reaction) as a threat, which may then get in the way of sensitive behavior in the moment. Notably, for mothers that showed less RSA reactivity, the relation between maternal PTSS and lower maternal sensitivity was not present, suggesting that continued engagement of the parasympathetic nervous system during caregiving may be protective even in the presence of high levels of PTSS. Prior research in older children showed that maternal PTSS was related to more non-supportive parenting at lower levels of maternal RSA reactivity, not higher RSA reactivity (Gurtovenko & Katz, 2020) and understanding how these relations shift over the course of development is crucial moving forward. Clearly establishing how flexible use of parasympathetic activation can aid parenting, while being mindful of overactivation and disconnection, needs further study during this vital period of early postpartum.

In line with prior research showing that RSA at rest and in response to stress are strongly related (Beauchaine, 2001), resting RSA and RSA reactivity were negatively correlated in our sample; mothers with higher resting RSA also showed greater reactivity during the caregiving task. Our convergent findings across moderation models suggest that PTSS is particularly disruptive to maternal sensitivity in caregiving for mothers who show these physiological patterns. Specifically, among trauma-exposed postpartum mothers, PTSS may interact with overregulated or excessive physiological responding to undermine sensitivity toward their infant.

There are many potential clinical implications of these results. First, PTSS in postpartum remains largely overlooked (Canfield & Silver, 2020) and our findings add to the growing research showing that maternal PTSS should be identified and treated in postpartum to reduce intergenerational effects of PTSS through early parenting behavior. Efforts are needed at system levels (e.g., obstetrics clinics) to increase access to PTSS screening and referral processes (Powers et al., 2020) and expand evidence-based treatment options to address the large gaps in available care for PTSS among postpartum women (Yildiz, Ayers, & Phillips, 2017). Second, evaluating the presence of physiological reactivity in postpartum women with PTSS may also be helpful to target interventions toward mothers at higher risk. Trauma-focused treatment, the recommended first-line approach for PTSD treatment (Watkins, Sprang, & Rothbaum, 2018), may adequately reduce these symptoms on its own. Additionally, emotion regulation skills training that teach strategies for addressing dissociation or reactivity (e.g., grounding) may be helpful to enhance autonomic functioning and reduce potential physiological impacts on parenting behavior in the context of PTSS. One example is Skills Training in Affective and Interpersonal Regulation (STAIR) (Cloitre, Koenen, Cohen, & Han, 2002), which can be used as a stepped care model with trauma-focused treatment (M. Hassija & Cloitre, 2015). A skills group designed specifically for perinatal women, such as mothers and babies (Tandon et al., 2022), is another option to consider and may be more approachable for some mothers who may not be interested in engaging with trauma content but are willing to learn strategies that help combat maternal stress (Tandon et al., 2021). Given so few studies have tested interventions for PTSS in postpartum, more research into these options is needed and should include physiological and parenting behavior measures to understand the potential impacts of treating PTSS on these mechanisms of interest and its relation to infant outcomes.

There are several strengths of this study, including a focus on the earliest stages of infancy, inclusion of a diverse sample of trauma-exposed mothers and infants, and inclusion of any trauma types for mothers versus restriction to one trauma type (e.g., intimate partner violence). However, there are important limitations that must also be acknowledged. First, the sample size was modest and may have been underpowered to detect small effects, although it was adequate for detecting medium to large effects consistent with prior research on our variables of interest. Next, the focus of this study was on maternal behavior and responses, and we know that understanding these associations in other caregivers is important and should be included in future studies. Additionally, we used a self-report measure of PTSS rather than a diagnostic interview. Although the PCL-5 is widely used and demonstrates strong psychometric properties (Weathers et al., 2013), self-report measures can be more susceptible to situational distress or item misinterpretation. As such, they may overestimate symptom severity and cannot confirm diagnostic status. Future research incorporating a clinician-administered diagnostic interview, such as the CAPS-5, during the behavioral visit will be important for validating these findings in the context of PTSD diagnosis. Finally, there may have been variability in how infants responded to the task, which could have affected the mother’s physiological and behavioral response. While infant distress was not coded in the present data, available data on infant negativity was examined and infant negativity was not correlated with our variables of interest.

In conclusion, we found that autonomic function plays a unique role in shaping how PTSS negatively impacts maternal sensitivity in the first 3 months postpartum. Both high baseline physiological regulation and heightened reactivity to caregiving strengthen the PTSS-low maternal sensitivity connection. Given that this is the first study to explore these effects in the earliest stages of development, more research is needed to further understand the interplay between PTSS and physiological regulation in predicting maternal sensitivity, and ultimately infant and child health outcomes.

## Supporting information

Powers et al. supplementary material Powers et al. supplementary material

## Supplementary material

The supplementary material for this article can be http://doi.org/10.1017/S0033291725102432.
