# Supplementary material for: Maternal PTSD symptoms and sensitivity during caregiving in early postpartum: The moderating role of resting and reactive RSA in a trauma-exposed sample
Source: Psychol Med. 2025 Nov 11;55:e339. doi: 10.1017/S0033291725102432 (PMC12775881; doi:10.1017/S0033291725102432)
Supplement: Powers et al. supplementary material [file S0033291725102432sup001.docx]

**Supplementary Materials**

**Supplemental Method**

**Coding of Infant Negativity to Parent**

Infant negativity to parent was coded based on video recordings of the maternal-infant interaction during the caregiving task and considered as a potential covariate. Infant negativity reflects how upset the infant appeared during the interaction, with a focus on behaviors or negative emotions directed toward the mother. Infant negativity to parent coding was adapted from the NICHD Study of Early Child Care, which has demonstrated similar psychometric properties across ethnoracially diverse dyads (Engel et al., 2021; Fuligni & Brooks-Gunn, 2013). Infant negativity was rated on a 1 to 4 scale, from 1 (*no negativity*) to 4 (*high negativity*) toward parent. Raters were blind to family characteristics and did not engage in study visits. Raters were trained and met reliability prior to rating interactions for this study.

**RSA Data Acquisition and Processing**

ECG was recorded using a modified Lead II configuration with three spot Ag/AgCl electrodes, placed at the bottom-most right rib, one on the left clavicle, and a ground electrode on the bottom-most left rib. Impedance cardiography was recorded with four spot Ag/AgCl electrodes, two used as receiving electrodes on front of the body (below the sternum, at the top of the sternum near the jugular notch), and two as sending electrodes on the participants’ back (1 inch lower and higher, respectively). Physiological data (ECG and impendence were acquired using MindWare Biolab version 3.0 (Mindware Technologies, Gahana, Ohio), a max sampling rate of 500 Hz for transmission of data wirelessly from the mobile unit was used. Cardiac impedance yielded an estimate of respiration, which was used to ensure that respiration frequency was within the range required for RSA calculation. Psychophysiological data were collected, filtered, extracted, and scored using Mindware software. RSA was derived using spectral analysis of the interbeat intervals (IBI) recorded through ECG, using fast Fourier transformation on the IBIs, which were detrended, centered, and tapered with a Hamming window, to decompose heart rate time series into component frequencies. High-frequency heart rate variability was extracted to quantify RSA.

**Supplemental Results**

Infant negativity distribution was highly skewed with 69.9% (*n =* 51) of infants rated as no negativity, 13.7% (*n =* 10) as minimal negativity, 4.1% (*n =* 3) as moderate negativity and 12.3% (*n =* 9) as high negativity. Thus, the infant negativity variable was recoded to a binary 0/1 variable reflecting 0 (no negativity; 69.9%, *n* = 51) and 1 (some negativity; 30.1%, *n* = 51). The recoded infant negativity variable was not correlated with total PCL-5 scores, *r* = .18, *p* = .13, maternal sensitivity, *r* = -.13, *p* = .26, baseline RSA, *r* = -.13, *p* = .27, or RSA reactivity, *r* = .15, *p* = .23.
